# Effects of Hybridized Synthetic Fibers on the Shear Properties of Cement Composites

**DOI:** 10.3390/ma13225055

**Published:** 2020-11-10

**Authors:** S.M. Iqbal S. Zainal, Farzad Hejazi, Farah N. A. Abd. Aziz, Mohd Saleh Jaafar

**Affiliations:** 1 Department of Civil Engineering, Universiti Putra Malaysia, Selangor 43400, Malaysia; iqbal.zainal@ums.edu.my (S.M.I.S.Z.); farah@upm.edu.my (F.N.A.A.A.); msj@upm.edu.my (M.S.J.); 2 Department of Civil Engineering, Universiti Malaysia Sabah, Sabah 88400, Malaysia

**Keywords:** forta fibers, hybrid fiber-reinforced concrete, fiber-reinforced concrete, shear energy, shear strength, hybridization synergy

## Abstract

The use of fibers in cementitious composites yields numerous benefits due to their fiber-bridging capabilities in resisting cracks. Therefore, this study aimed to improve the shear-resisting capabilities of conventional concrete through the hybridization of multiple synthetic fibers, specifically on reinforced concrete structures in seismic-prone regions. For this study, 16 hybrid fiber-reinforced concretes (HyFRC) were developed from the different combinations of Ferro macro-synthetic fibers with the Ultra-Net, Super-Net, Econo-Net, and Nylo-Mono microfibers. These hybrids were tested under direct shear, resulting in improved shear strength of controlled specimens by Ferro-Ultra (32%), Ferro-Super (24%), Ferro-Econo (44%), and Ferro-Nylo (24%). Shear energy was further assessed to comprehend the effectiveness of the fiber interactions according to the mechanical properties, dosage, bonding power, manufactured material, and form of fibers. Conclusively, all fiber combinations used in this study produced positive synergistic effects under direct shear at large crack deformations.

## 1. Introduction

Although the normal-strength concrete is strong in compression, it is brittle and requires the support of steel reinforcements in order to prevent shear, tensile, and flexural failure. This poses problems for structures in seismic-prone regions since more steel reinforcements are needed to efficiently manage the crack zones. Consequently, the incorporation of a large amount of steel reinforcements would result in certain complications, such as steel congestion, improper concrete compaction, labor-intensive work, and increased project costs. In order to address these complications, the incorporation of fibers in cementitious composites is suggested to utilize their fiber-bridging effect and improve the mechanical properties of the structures [1].

To date, four different types of fibers are available for use, namely, steel (Type I), synthetic (Type II), glass (Type III), and natural (Type IV) fibers [2]. Steel fibers are the most frequently used fibers in the market but the current advances in textile technology have shifted towards synthetic fibers due to their high availability, rapid manufacturing, lower cost, and versatile applications. Furthermore, several companies have already developed macro-synthetic fibers that can replace the secondary steel reinforcements in concretes [3].

However, fiber-reinforced concretes (FRCs) remain bounded by the crack-zone limitation because a singular fiber can only address one specific type of crack, depending on the type of fiber used. This limitation can be addressed through hybridization between two distinct fibers. Studies have demonstrated the practicality of using HyFRC with the effective utilization of both macro- and micro-fibers in their crack-bridging capabilities. The use of fibers in cementitious composites is influenced by several parameters, such as the length, shape, aspect ratio, dosage, and materials [4]. The drawback of fiber hybridization is that there are no primary mixing guidelines, as the process mainly involves adapting normal concrete design mixes and observing any changes in the mechanical properties during the hardened stage. Fiber hybridization is based on preliminary observations and the need for improvement, rather than relying on the existing standards.

For instance, Walton and Majumdar [5] investigated concrete using asbestos, polypropylene, natural, glass, and carbon fibers. The study found that the combination of organic and inorganic fibers yielded positive synergistic effects, which improved the mechanical properties of concrete. On the other hand, Banthia and Sheng [6] performed an investigation on carbon and steel fibers and reported increased strength of concrete with every addition of steel fibers and increased toughness of concrete with the addition of carbon fibers. Meanwhile, Soroushian et al. [7] proved the effectiveness of combining polyethylene and polypropylene fibers in increasing the performance of concrete in terms of impact loading, flexural strength, and toughness. In another study, Banthia and Soleimani [8] investigated the hybridization of steel, carbon mesophase pitch-based, carbon isotropic pitch-based, and polypropylene fibers, and observed that carbon isotropic pitch-based fibers produced better HyFRC than the carbon mesophase pitch-based fibers.

Kotecha and Abolmaali [9] reported that the use of polypropylene macro-synthetic fibers significantly increased the ultimate strength and post-cracking behavior of deep beams. Babafemi and Boshoff [10] studied the pull-out response of polypropylene macro-synthetic fibers in concrete and concluded that the bond between fibers and concrete is non-linear. Besides that, Parra-Montesinos et al. [11] tested beam–column-reinforced concrete made from polyethylene fibers and found that the specimens performed satisfactorily under shear reversals with excellent damage tolerance. Similar outcomes were also observed by Roesler et al. [12], where the addition of polypropylene and polyethylene fibers modified the failure behavior of concrete by effectively distributing the loads. Xu et al. [13] conducted a hybridization of steel and polypropylene fibers and observed that the fiber-hybridization produced improved tensile behavior of plain concrete. In another study, Altoubat et al. [14] hybridized polypropylene with polyethylene fibers, which changed the mode of failure in shear from brittle to ductile for concrete without stirrups. On the other hand, Ramanalingam et al. [15] hybridized polyvinyl alcohol with steel fibers, which improved the performance of cementitious matrix in post-crack behavior. Navas et al. [16] conducted an experimental study on the shear behavior of reinforced concrete with polypropylene macro-synthetic fibers, and the findings showed that the macrofibers serve as an effective mechanism of shear transfer in cementitious composites.

Meanwhile, Banthia and Gupta [17] studied the various forms of polypropylene fibers and found that fibrillated polypropylene fibers were more effective than its monofilament counterpart in reducing plastic shrinkage cracks. Greenough and Nehdi [18] studied various forms of steel and polypropylene fibers and concluded that the fiber length does not provide significant improvement but fiber material does. However, Sun et al. [19] found that the combination of various lengths of steel fibers reduced shrinkage strains. Ma et al. [20] hybridized different types of steel fibers which improved the mode of failure of fibrous concrete under shear. Jongvivatsakul et al. [21] studied various fiber materials, specifically steel, polypropylene, polyvinyl alcohol, and polyethylene fibers, and discovered a significant increase in the shear capacity of beams for all fiber materials. Zhang et al. [22] added polypropylene fibers to cementitious composites and found improvements in the shear carrying capacity for both beams (with and without shear reinforcements). Joshi et al. [23] studied the efficiency of steel and synthetic fibers on beams under flexure shear. Both fibers improved the post-cracking and ductility of concrete, with steel fibers being more efficient than the macro-synthetic fiber. Yew et al. [24] examined the effects of the twisted-bundle form of polypropylene fibers and found positive enhancement in the properties of oil palm shell concrete. Additionally, Zainal et al. [25] hybridized multiple types of macro- and micro-synthetic fibers with different fiber properties, which resulted in improved compressive and tensile behavior in concrete.

Overall, previous studies have demonstrated that synthetic fibers do impart strength and toughness in shear to concrete. Engineered cementitious composites with embedded fibers have been used in structural components to distribute the stress more efficiently and increase the strain capacity of reinforced concrete, resulting in reduced shear steel reinforcements. Although macro-synthetic fibers possess lower modulus of elasticity and may not be able to provide a comparable shear strength as opposed to steel fibers, the desired ductile properties imposed on concrete that delay the brittle failure and reduce excessive loads inflicted towards the stirrups are preferred for use in seismic-prone regions.

## 2. Hybridization of Synthetic Fibers

The current study attempted to increase the shear strength and toughness of concretes and reduce the number of shear steel reinforcements required in the narrow spaces of structural members located in seismic-prone areas, such as for the case of beam–column joints. Accordingly, one of the main steel reinforcements that contribute to steel congestion in reinforced concrete (RC) structures is the stirrup. The elimination or reduction in the quantity of stirrups is expected to address the highlighted complications more efficiently through the use of HyFRC. 

The hybridization of macro-synthetic fibers with other microfibers was selected in the effort to eliminate the dependency of using steel fibers as the primary load-bearing fibers. The benefits of using synthetic fibers are that these fibers are more economical than steel fibers and achieve a similar degree of reinforcement to concrete [26]. Synthetic fibers are also non-corrosive in nature, as opposed to steel fibers, in which studies have proved that corrosion deteriorates the performance of concrete structures. In addition, the production of synthetic fibers also significantly reduces the carbon footprint compared to the production of steel fibers [27,28].

**Figure 1:**
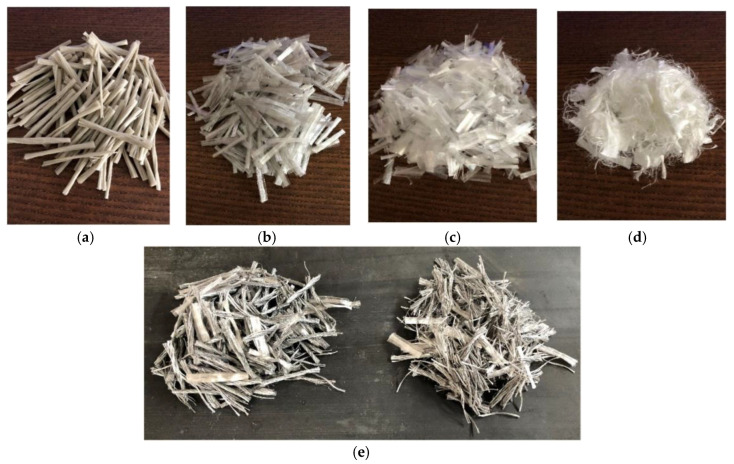
All the fibers used in this study which consists of the (**a**) Ultra-Net, (**b**) Super-Net, (**c**) Econo-Net, (**d**) Nylo-Mono microfibers and the (**e**) Ferro macrofibers.

Furthermore, this study exclusively focused on synthetic fibers manufactured from FORTA Corporation (Grove City, PA, USA) with varying parameters, such as fiber-form, optimal volume fraction of fibers, tensile strength, fiber length, bonding power, manufactured materials, and fiber-class, which indirectly affect the shear strength of cementitious composites. For this study, the secondary fibers were the Ultra-Net, Super-Net, Econo-Net, and Nylo-Mono microfibers. Meanwhile, Ferro was the primary fiber in the hybrid mix and dual lengths were used to maximize post-crack performance under shear, as short and long fibers have specific roles during cracking that are known to benefit HyFRC [29]. All of the fibers used in this study are shown in Figure 1.

As shown in Table 1, the selected fibers in this study have different fiber characteristics. The different parameters affect the synergistic effects of the fiber combinations and consequently, the shear strength and toughness. The different fiber characteristics that affect the performance of concrete can be categorized into several categories:

1. Fiber size. In this study, two different fiber classes were hybridized to produce macro–micro-fiber-mix combinations. Each fiber class is subjected to bridge the different levels of cracks during shear failure. The influence of other parameters may affect the conventional fiber-bridging capabilities of the macro–micro combination, which were further investigated.
2. Fiber length. Fiber length corresponds to effective fiber-bridging capabilities. Shorter fibers lead to higher fiber count per density, resulting in enhanced concrete performance through multi-cracking. Longer fibers tend to improve the post-cracking behavior by enabling a quasi-brittle mode of failure. The effects of different fiber lengths from different types of fibers used were then further studied.
3. Volume fraction of fibers. The different volume fractions of fibers were used to evaluate the possible variations in shear strength for the macro–micro-fiber combinations with increasing fiber dosage, as well as to determine an optimal macro–micro-fiber combination.
4. Bonding power. The intensity of bond stress between the fibers and their surrounding cementitious matrix determines the fiber failure, whether by fiber-breakage or effective pull-out failure. The mode of failure influences the overall shear strength and toughness of HyFRC.
5. Manufactured materials. The materials considered in this study were polypropylene and polyethylene for the primary fibers and polypropylene and nylon for the secondary microfibers. Additives were added to differentiate the fibers from other synthetic fibers. Each material has varying tensile strength, which is directly correlated with the fiber mode of failure.
6. Fiber form. The manufactured form has a direct relationship with the bonding power, which provides different anchorage intensities during fiber-bridging. The anchorage intensity for each fiber in improving the shear strength of HyFRC was assessed.

Table 2 shows the developed hybrid-mix designs with one plain concrete and five controlled FRCs—the Ultra-Net Control (UC), Super-Net Control (SC), Nylo-Mono Control (NC), and Ferro Control (FFC). The designation for each HyFRC mix-design is represented by the first letter of its constituent fiber followed by the number that represents the volume fraction used for the respective fiber. Each of the 22 mix-designs have specific designations which will be used interactively throughout this paper. The performances in terms of shear strength and toughness of the developed HyFRC were compared against the conventional non-fibrous concrete and single-fiber FRC to measure the improvements in shear strength, toughness, and hybridization synergies. The mix combinations, specifically F6U3, F6S3, F6E3, and F6N3, in this study had similar fiber dosage as their controlled microfiber counterparts (UC, SC, EC, NC, and FFC) for direct comparison in order to assess the hybrid fiber synergistic effects.

Additionally, the details of the concrete design in this study were as follows: (1) cement = 409 kg/m^3^, (2) water = 225 kg/m^3^, (3) sand = 836 kg/m^3^, (4) 10 mm coarse aggregates = 302 kg/m^3^, and (5) 20 mm coarse aggregates = 604 kg/m^3^. Portland cement Type II was used from various sources. No superplasticizers were used with HyFRC in order to observe the initial effect of hybridization on cementitious composites for the normal concrete mix-design. Meanwhile, the shear strength of the developed HyFRC was tested using the Japanese Standard Specification for Concrete Structures JSCE-G 553-1999 [30]. A total of three 100 × 100 × 500 m specimens were produced for each design combination, totaling up to 66 prisms for the entire study. In particular, these prisms were de-molded after 24 h and cured for 28 days in a water tank prior to the direct shear test.

**Figure 2:**
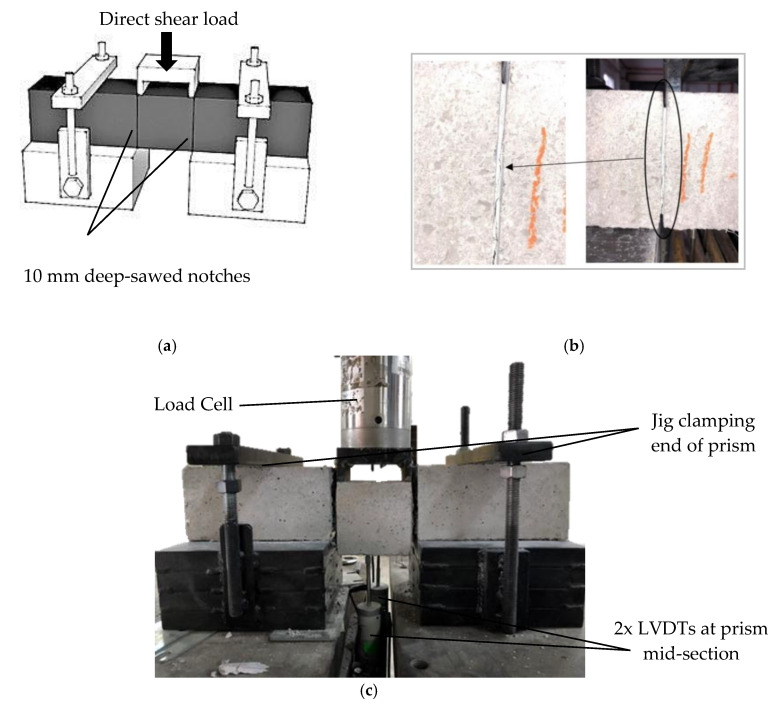
Photos illustrating and displaying the (**a**) test set-up according to the JSCE-G 533-1999 test standard, (**b**) notches sawed to pre-define crack propagation, and (**c**) direct shear testing of the specimens.

Figure 2a shows the position of linear variable displacement transducers (LVDT) during the direct shearing test. The jigs and test set-up were fabricated and positioned according to the recommended specifications. Two LVDTs were clamped and aligned under the middle of the prism using magnetic holders that were firmly attached to the wide steel base. A hydraulic hand-pump was also used to apply a steady rate-of-load at 1 kN per increment. The 100 kN load cell was connected to a data logger along with two LVDTs to record the imposed shear loads and the corresponding prism mid-deflections. The following formula to calculate the shear strength of the developed HyFRC was used in this study:

(1) $$\tau \;=\;\frac{P}{2bh}$$

where *τ* denotes the shear strength (N/mm^2^), *P* denotes the maximum load obtained (N), *b* refers to the width of specimens obtained (mm), and *h* refers to the height of specimens obtained (mm).

According to the JSCE test standard [30], diverted cracks that are propagated away from the anticipated failure plan are to be discarded, but past studies demonstrated the difficulty to control the crack propagation along the failure plane [31]. Henceforth, in order to ensure a direct shear approach, two notches with the width measurement of 1 mm and depth measurement of 10 mm were sawed at each side (left and right sides) of the mid-span area to pre-define the crack propagation during the testing. Figure 2b presents the two notches that were prepared before the testing, whereas Figure 2c illustrates the overall test set up. In order to assess the effectiveness of the fiber hybridization in dissipating energy under direct shear, the area under the load-deflection curve was calculated to obtain shear energy. Subsequently, the synergistic effects from the energy absorption capacity were observed at different crack deflections of 1, 3, and 5 mm in order to gain a better understanding of the performance of the cementitious composites with combined fibers.

## 3. Results and Discussion

This study aimed to improve the shear-resisting capabilities of cementitious composites through the hybridization of multiple synthetic fibers. The application was directed at RC structures located in the seismic-prone regions, where critical areas such as the beam-column joints are limited by narrow spaces due to steel congestion. For this study, the JSCE-G 553-1999 test standard was used to assess the shear strength and load-deflection curves for the developed HyFRC. The toughness or energy absorption capacity was computed to further assess the dissipated energy under direct shear. With that, the synergistic effects between the hybridized fibers were assessed based on the shear energy in order to gain a better understanding on the interaction between the combined fibers within the cementitious matrix towards improving shear strength and toughness.

### 3.1. Density

**Figure 3:**
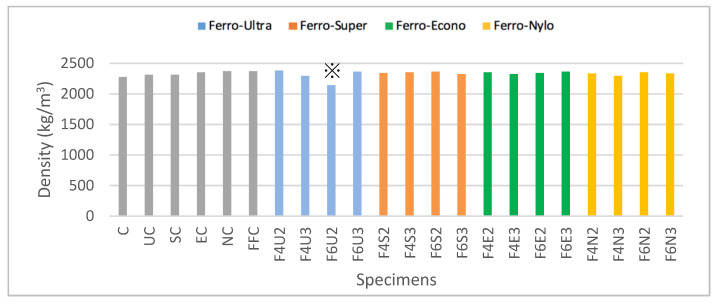
HyFRC densities. (※ = irregular density).

Figure 3 shows the recorded average density for all HyFRC specimens, which revealed that F6U2 recorded the lowest density at 2143 kg/m^3^ whereas NC recorded the highest density at 2372 kg/m3. The percentage difference between the lowest and highest values of density for all mix-designs was found at 10.41%. The recorded value for the F6U2 density is the lowest, possibly due to the entrapment of excessive air or because of improper mixing during the material preparation stage. The F6U2 mix-designs were marked with the symbol ※ for further evaluation on the possible impact towards the HyFRC shear strength results. Nevertheless, the recorded values of density for other HyFRC specimens were not significantly different, thus suggesting consistent dispersion of materials during the casting. 

### 3.2. Shear Strength

**Figure 4:**
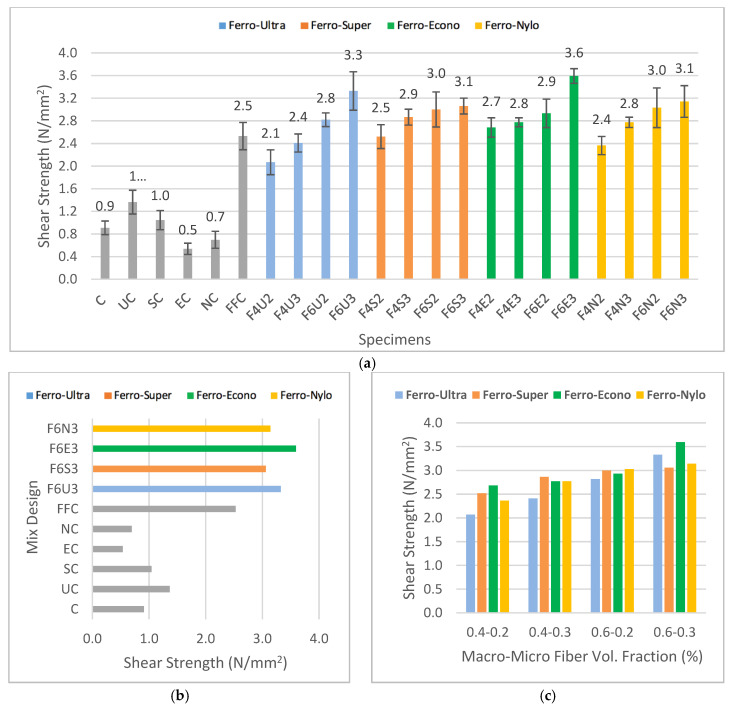
The shear strength of (**a**) developed HyFRC with error bars, (**b**) control vs. HyFRC specimens, and (**c**) HyFRC categorized by fiber dosage.

Figure 4a presents the shear strength for all HyFRC specimens, which revealed that Ferro-Ultra hybrids between the F4U2 and F6U3 designs recorded the highest percentage difference between the lowest and highest values of shear strength at 44.44%, followed by Ferro-Econo hybrids of between F4E2 and F6E3 designs (28.57%), Ferro-Nylo hybrids of between F4N2 and F6N3 designs (25.45%), and lastly, Ferro-Super hybrids of between F4S2 and F6S3 designs (21.43%). The combination of Ferro and Ultra-Net fibers produced the most significant increase in shear strength with the increase in fiber dosage. This was followed by the combinations of Ferro with Econo-Net, Nylo-Mono, and Super-Net fibers, respectively. The recorded improvements for these combinations were found within a similar range, with an average of 25.15% improvement in shear strength with the increase in fiber volume fraction. The increasing trend of shear strength in this study proved the effectiveness of hybridization in bridging shear cracks with the increase in the amount of macro- and micro-sized fibers used. This is consistent with the findings from previous studies [20,32].

Meanwhile, the plain concrete recorded a shear strength of 0.9 N/mm^2^. When it comes to the controlled specimens, FFC recorded the highest shear strength of 2.5 N/mm^2^. In other words, Ferro macro-synthetic fibers in hybridization endured the highest shear load during the fiber-bridging process. In addition, UC produced shear strength of 1.4 N/mm^2^, while EC and NC resulted in weakened shear strength of 0.5 and 0.7 N/mm^2^, respectively. The addition of single-fiber Econo-Net and Nylo-Mono in cementitious matrix reduced the shear capability of conventional concrete by 44.44% and 22.22%, respectively. On the other hand, the addition of Ultra-Net (by 55.56%) and Super-Net (by 11.11%) microfibers increased the shear strength of conventional concrete.

As shown in Figure 4b, the hybridization of macro–micro-fibers showed improvement in shear strength. Although the EC- and NC-controlled FRC were found to reduce the shear strength of concrete, the addition of Ferro macrofibers in the hybrids improved the overall shear strength, even more than the FFC mix-design. These results demonstrated the effectiveness of using different types of fibers in cementitious composites to bridge different levels of crack. The use of different types of fibers have been reported to improve the crack-resisting capabilities in other studies as well [5,7].

Microfibers generally play a vital role in increasing the peak strength of concrete by strengthening the monolithic integrity of concretes and bridging micro-cracks as well as preventing these small cracks from coalescing and propagating in singularity to larger cracks [33]. A microfiber-only FRC would be stronger against crack and would require significantly higher loading to arrive at its peak stress. However, without the use of any macro-sized fibers in concrete, brittle crack failure would occur after the peak stress threshold limits are exceeded. The micro-cracks localize and propagate into macro-sized cracks at a rapid pace without fully utilizing the fiber-bridging effect at the micro-level. In fact, all fiber combinations under study were found to improve shear strength more than the single-fiber FRC. The F6U3 mix-design held securely in cementitious matrix (or generally known as “pegging”) during shear cracking. The combination of FFC and UC in this study resulted in the largest improvement in shear strength per fiber volume. This led to a conclusion that fibrillated fibers produce a higher modulus of elasticity when intertwined and can be more effective in resisting the dowel action under shear [14]. In fact, Soufeiani et al. [34] have deduced that the shape of fibers plays an important role in improving the performance of concrete due to the better bonding features between fibers and cement composites.

Nevertheless, the hybridization of FFC with fibrillated microfibers, such as SC, EC, and UC, resulted in the best shear strength at every fiber dosage tier, as shown in Figure 4c. Although the improvement per fiber dosage for the FFC-SC and FFC-EC hybrids were not as significant as the FFC-UC hybrids, the produced shear strength for fibrillated polypropylene microfibers was still the highest at every volume faction tier compared to monofilament virgin nylon used for the FFC-NC hybrids.

Adding to that, the synthetic fibers used are classified according to their bonding power, or in other words, the interfacial bond stress between the fibers and their surrounding cementitious matrix. Four types of bonding power were considered in this study, namely (1) extra heavy-duty, (2) heavy-duty, (3) medium-duty, and (4) low-duty. The combination of heavy-duty FFC and extra heavy-duty UC yielded the least shear strength due to the excessive bond-stress imposed by the hybrid fiber combinations against concrete during fiber pull-out. This led to fiber-breakage during the direct shear loading. A moderate fiber bonding power by the SC, EC, and NC hybrids resulted in a more gradual and effective pull-out. This type of failure dissipates more energy and provides additional strength to the HyFRC [35]. This can be seen by the slight increase in shear strength for the fibers, as compared to the Ferro-Ultra hybrids in the range of 0.4–0.2%, 0.4–0.3%, and 0.6–0.2% fiber volume fraction.

**Figure 5:**
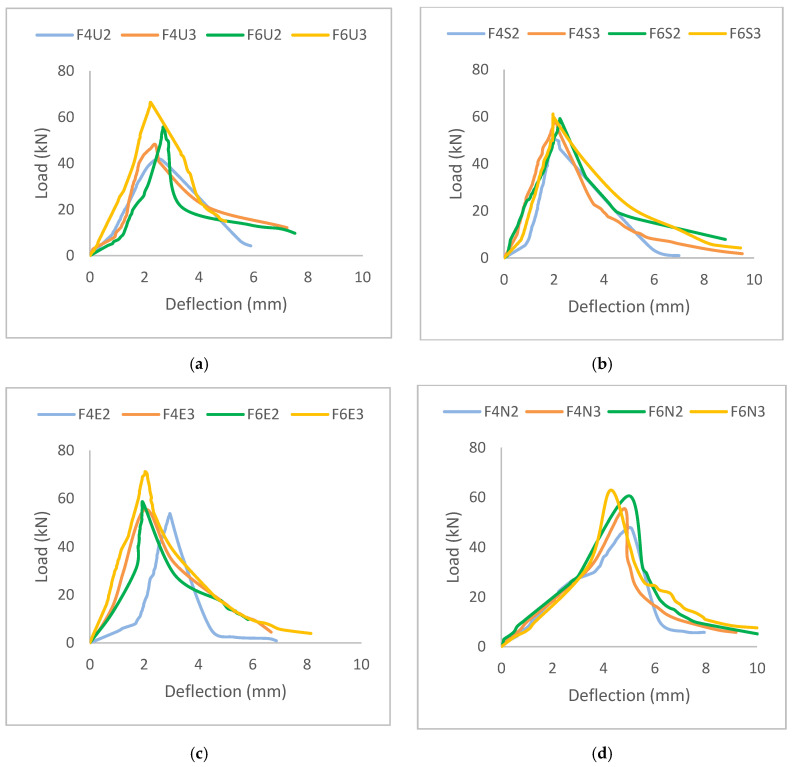
The load-deflection curve for (**a**) Ferro-Ultra, (**b**) Ferro-Super, (**c**) Ferro-Econo, and (**d**) Ferro-Nylo hybrids under direct shear.

**Figure 6:**
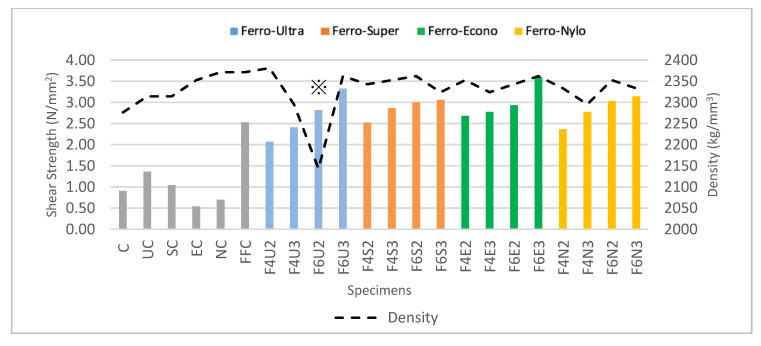
Influence of density on HyFRC shear strength (※ = irregular density).

The behavior of each HyFRC specimen under direct shear can be observed in Figure 5. As illustrated in the graphs, all hybrids reached their peak shear strength within the deflection range of 2 mm, except for the Ferro-Nylo hybrids (at approximately 5 mm). Additionally, all developed HyFRC specimens showed quasi-brittle characteristic with a strain-softening mode of failure under the direct shear. Similar findings have also been reported in other studies [31,36]. Figure 6 superimposes the density on the shear strength of all HyFRC specimens, which revealed that a proper, consistent mix was achieved during the casting of the HyFRC with no effect on the shear strength results. The irregular density observed for the F6U2 design displayed the same increasing pattern in shear strength with increase in fiber volume fraction like the rest of the Ferro-Ultra hybrids.

### 3.3. Shear Energy

**Figure 7:**
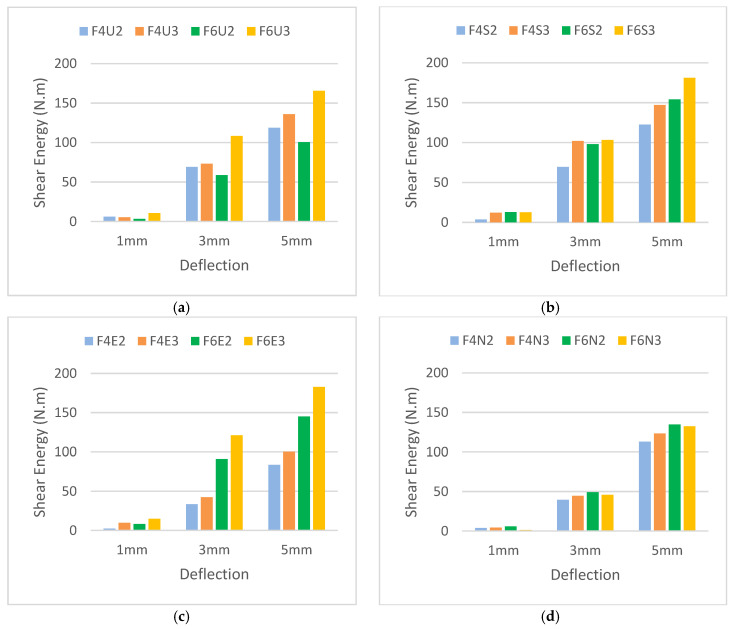
The shear energy absorption capacity for the (**a**) Ferro-Ultra, (**b**) Ferro-Super, (**c**) Ferro-Econo, and (**d**) Ferro-Nylo hybrid.

The ability of the developed HyFRC to absorb shear energy was measured at mid-span prism deflection of 1, 3, and 5 mm in order to observe the effectiveness of fiber hybridization under direct shear. Figure 7 shows the energy absorption capacity for all fiber combinations used in this study. Overall, a direct correlation was found in this study with previously conducted studies [21,22], where the increase in fiber dosage corresponded to the increase in shear energy. Higher mid-span deflection showed higher energy absorption capacity for all fiber combinations, thus suggesting effective fiber-bridging between the hybridized fibers under shear. Only the F6U2 design showed lower shear energy in the category of Ferro-Ultra combinations due to the instability of the prism during cracking. A higher quantity of fibers was present on one side of the prism, which resulted in the other side resisting the shear loads more. 

**Figure 8:**
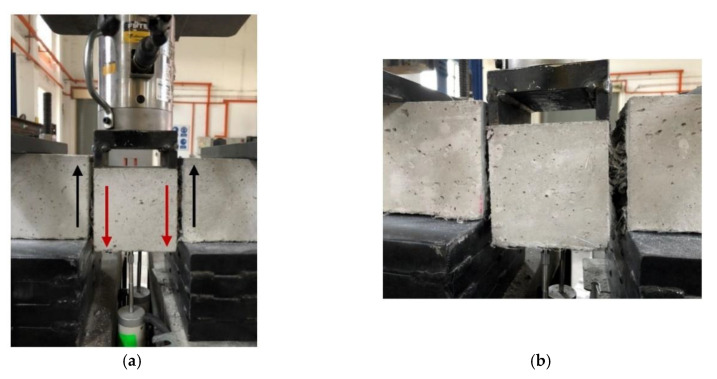
Experimental photos showing the (**a**) in-plane shear (mode II fracture) failure and (**b**) specimen instability due to higher fiber count on the right-hand side of the prism.

Accordingly, Figure 8a illustrates the direct shear imposed on the prism directly on the mid-span sawed notches, while Figure 8b captures the fiber count instability for the F6U2 specimen. The Ferro macrofiber plays a major role in enhancing the energy absorption capacity of HyFRC. The frictional fiber pull-out during cracking dissipates energy, thus allowing the HyFRC to absorb more energy during shear [37]. Although the 38 mm Ferro has a lower aspect ratio than its 54 mm Ferro counterpart, it exhibited a larger fiber count in the given mix. These fibers can be easily dispersed in cementitious composite [38], which ensures a high percentage of fibers to be positioned at the line of crack propagation. Subsequently, the 54 mm Ferro fibers appeared to be more effective, as larger crack gaps started to widen where the fiber-bridging effects of 38 mm fibers were limited due to its length. These 54 mm fibers were effective in anchoring the widening interfacial crack surfaces during the in-plane shear (Mode II) failure [29].

**Figure 9:**
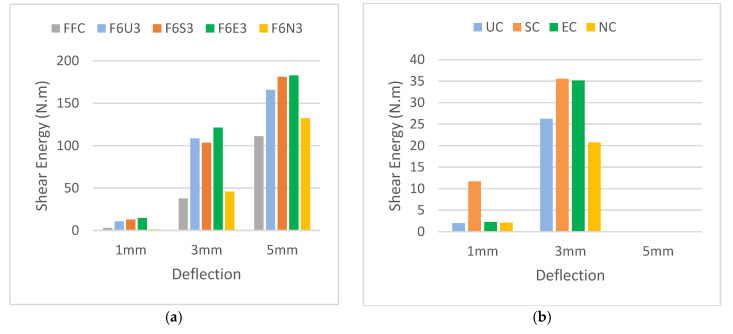
The shear energy comparison for (**a**) HyFRC specimens and (**b**) single-fiber FRC specimens.

Meanwhile, referring to Figure 9a, for polypropylene micro-sized fibers, the 38 mm microfibers appeared to absorb more energy than their 54 and 19 mm counterparts because longer fibers are typically used to improve the post-cracking behavior of cementitious composite. This was proven to be useful for the Ferro macrofibers. However, for the Ultra-Net, Super-Net, and Econo-Net microfibers, their length-benefits were not as useful in arresting micro-sized cracks but their high fiber count was found more useful in incurring strain-hardening effects during the micro-cracking stage. This is consistent with the research published by Lawler et al. [39] and Chasioto et al. [40]. Both EC and SC fibers had the same length of 38 mm but the difference in surface-form was found to exhibit more significant improvement to the overall energy absorption capacity of HyFRC. The 19 mm NC fibers in this study had the shortest fiber length but showed the poorest performance in the shear energy produced for the category of Ferro-Nylo combinations. This can be attributed to the dependence of NC nylon materials on bonding power with the surrounding concrete, rather than the physical tensile length of the fiber [41]. On the other hand, unlike the 38 mm fibrillated polypropylene fibers, the monofilament form was unable to provide strong anchoring to the concrete. Basically, it can be deduced that the NC fibers produced lesser friction during the pull-out and were unable to effectively synergize and provide additional support for the Ferro macrofibers during fiber failure under direct shear.

Figure 9b presents the energy absorption capacity of the controlled single-fiber specimens. No shear energy was recorded for all single-fiber FRC at 5 mm because the load-deflection curves fractured at around 3 to 4 mm deflection. Hence, no shear energy data can be obtained at that deflection. The results demonstrated that the synthetic microfibers imparted minimal shear toughness to the HyFRC, as most of the fiber-bridging effects in shear for the micro-FRC ended at prism deflection of 3 mm. In other words, their physical tensile limit was somehow restricted to a lower deflection increment. After all, the addition of microfibers in hybrid is often ineffective, as the increase in the peak strength leads to additional hardening of concrete [39]. The increase in interfacial bonds from the hardening resulted in undesirable fiber-breakage during the crack-bridging process, as the tensile limit of the synthetic fibers was exceeded. 

However, it was also observed that several HyFRC specimens succeeded in increasing the energy absorption capacity higher than that of singular-Ferro FRC. The mechanics of the interaction between the macro–micro-fibers within the cementitious composites largely contributed to the increase in the shear performance of HyFRC. As previously stated, the addition of microfibers in the mix often resulted in fiber-breakage of macro-sized fibers during the post-cracking stage due to the excessive hardening of concrete. All UC, SC, EC, and NC microfibers in this study imparted shear toughness, extending their fiber-bridging capabilities into macro-zones beyond the intended peak stress enhancement capabilities at the micro-level. In essence, the synthetic microfibers in this study provided additional resistance to the HyFRC during the post-cracking, up to the point of exceeding their tensile limit. This has been proven in various experimental testing involving fibers [15,42,43]. The supplementary support at the macro-level cracks was found to eliminate the abrupt shear loads exerted to the Ferro fibers and reduce the excessive shear force, thus allowing a more progressive frictional pull-out of the macro-synthetic fibers to bridge the widening large crack gaps and dissipate the shear energy. 

**Figure 10:**
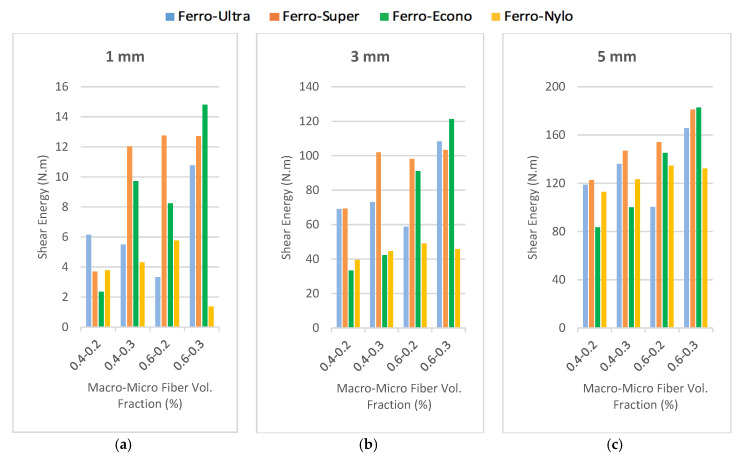
HyFRC comparison by fiber dosage tier at (**a**) 1 mm, (**b**) 3 mm, and (**c**) 5mm deflections.

Microfibers with physical form that enhance the bonding power with concrete, such as the UC, performed poorly compared to their other polypropylene counterparts, such as the SC and EC. The excessive bonding between the fibers and cementitious matrix appeared to prevent frictional pull-out and allow abrupt fiber-breakage. The EC and SC were able to impart more shear toughness to the hybrids because they were able to incrementally slide during the frictional tensile elongation due to their relatively weaker bonding power. This conclusion aligns with the study conducted by Okoli and Smith [44] as well as Bolander and Choi [45]. On the contrary, NC fibers are hydrophilic and absorb water during the mixing of fresh concrete. The excess water stored inside the fibers is available for hydration and hardens the concrete area surrounding the Ferro and Nylo-Mono fibers. The excess hydration increases the shear strength of the NC hybrids. However, due to the high load-intensity imposed on the specimens, the high shear energy from the rapid crack propagation results in fiber-breakage mode of failure for the NC microfibers. Consequently, this imposes an excessive strain on the Ferro fibers during the post-cracking and reduces the overall HyFRC toughness under shear [46,47]. The various performance outcomes of all HyFRC specimens at different crack levels are categorized according to fiber dosage tier (Figure 10).

## 4. Assessment of Results

### Hybridization Synergy

Assessing the effectiveness of the fiber combinations in cementitious composites involves the quantification of the synergistic effects via shear energy on the pre-defined crack deflections. It is necessary as it is difficult to physically observe any changes in the macroscopic behavior of the cementitious composites. The quantification of the synergistic effects illustrates the behavior of hybridized fibers and their overall effect on HyFRC. The following equation was adapted from a previous study by Banthia et al. [48] with respect to the purpose of this study:

(2) $$Synergy\;=\;\frac{SE_{hyb,\delta }}{SE_{a,\delta }+SE_{b,\delta }}-1,$$

where *SE_hyb,δ_* refers to the shear energy at specific deflections for HyFRC, *SE_a,δ_* refers to the controlled FRC for the selected macrofibers, and *SE_b,δ_* refers to the controlled FRC for the selected microfibers. 

The objective was to identify and compare the synergistic effects of the hybrid fibers and their improvement against the combined results of their individual FRC counterparts. The value of 1 represents the flexural toughness of normal concrete. A positive value (>0) implies positive synergy combination that contributes to the increase in the performance of shear energy. In that case, a negative synergy (<0) means a decrease in the performance of synergy energy for HyFRC, as compared to its single fibers, while zero synergy (= 0) indicates hybridization that does not increase or decrease the performance of shear energy for HyFRC.

**Figure 11:**
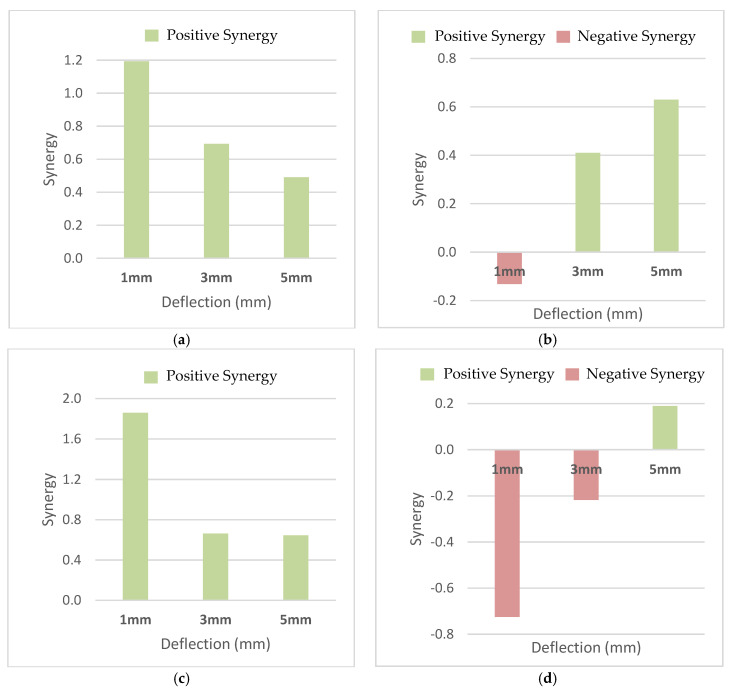
The synergistic effect in shear energy for (**a**) F6U3, (**b**) F6S3, (**c**) F6E3, and (**d**) F6N3 HyFRC.

**Figure 12:**
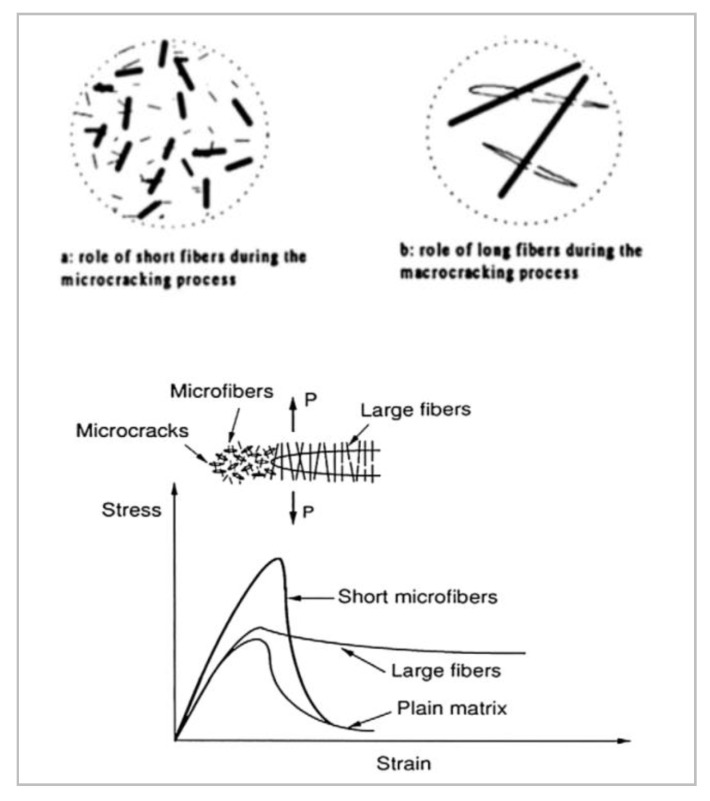
Role of macro- and micro-fibers during concrete fracture [49]. Reproduced from P. Rossi, Concrete International: 1982.

Accordingly, the F6U3, F6S3, F6E3, and F6N3 mix-designs were selected to represent the category of fibers for hybridization, namely the Ferro-Ultra, Ferro-Super, Ferro-Econo, and Ferro-Nylo fiber combinations. As shown in Figure 11, the F6U3 and F6E3 hybrids yielded positive synergistic effects for all deflections but showed signs of decreasing synergy energy with the widening mid-prism deflections. The decreasing synergistic effects may be attributed to the ineffective mode of failure of the Ferro macrofibers caused by the synthetic microfibers. The microfibers increase the peak shear stress of the HyFRC, but the improvement also escalates the likelihood of the Ferro macrofibers breaking. As illustrated in Figure 12, when the shear stress exceeds the yield strength of the HyFRC, cracks coalesce and propagate rapidly into the post-cracking region, which would lead to fiber-breakage of the Ferro macrofibers [39]. The abrupt cracking would make the Ferro macrofibers less effective in bridging the widening macro-sized cracks as fiber-breakage consumes less energy than debonding and pull-out failure. Nevertheless, the positive synergy effects illustrate the effectiveness of HyFRC in resisting in-plane shear failure from the fiber-bridging effects.

A higher amount of accumulated fibers at the interfacial surface at either side of the prisms would produce higher energy absorption capacity on one side and weaker energy absorption capacity on the other side, resulting in slight fiber-bridging instability. The produced synergy for the F6S3 design increased with the increase of deflection, thus implying an even dispersion of the hybridized fibers in the cement composites. In particular, the fiber combinations for the F6S3 design showed positive synergy effects at 3 and 5 mm but produced negative synergistic effects at 1 mm, although it was previously revealed that the controlled SC specimens were able to absorb a significant amount of shear at the crack deflection of 1 mm. This was the result of the fiber-bridging instability during the shear testing at that specific deflection. Although the differences in shear energy between SC and the other controlled specimens at 1 mm were substantial, the recorded shear strength showed no sign of exaggeration due to the fiber-bridging instability during the direct shear test on the prisms. 

The F6N3 fiber combinations produced negative synergies at 1 and 3 mm, which indicated that single-fiber FRC were more efficient in resisting direct shear, as compared to when they were combined. The yielded negative synergies were due to the hydrophilic properties of nylon; as water is absorbed during concrete mixing, stronger yet brittle concretes with higher peak stress can be produced. However, the hybridization synergies were found to increase with the increase in the mid-span prism deflections. Positive synergistic effects were produced at 5 mm, thus suggesting effective fiber combinations at larger crack openings. 

## 5. Conclusions

The main purpose of this study was to transform the concrete mode of failure from brittle to quasi-brittle in order to reduce the dependence on steel reinforcements for shear. This study exclusively developed cementitious composites with higher shear ductility and strength through fiber-hybridization using synthetic fibers from FORTA Corporation (Grove City, PA, USA). The testing was conducted using the JSCE-G 553-1999 test standard, followed by the assessment of shear energy and synergistic effects of hybridization. In this study, Ferro macro-synthetic fibers were combined with Ultra-Net, Super-Net, Econo-Net, and Nylo-Mono microfibers to examine the effects of bonding power, form, diametrical size, volume fraction, tensile strength, manufactured materials, and fiber length on the hybridization, and subsequently, shear-resisting capabilities. 

The obtained results of this study are subsequently summarized. Firstly, the Ferro macro-synthetic fibers in this study achieved pull-out mode of failure and effectively imparted shear strength and toughness to HyFRC as the primary load-bearing fibers, even when combined with selected microfibers. Furthermore, it was found that the increase in the volume fraction of Ferro fibers corresponded to the increase in shear strength and further improved the effectiveness of the cement matrix in absorbing shear energy.

Secondly, the direct shear imposed on the prisms in this study induced dowel-type movement on the fibers during the fiber-bridging effects. Hence, the effects of various microfibers on hybridization were limited by factors such as fibrillation type, bonding power, fiber length, and tensile length. Excessive bonding power and fibrillation-anchorage caused the fibers to exceed their tensile limit and breakage, while more moderate interfacial bonds promoted effective pull-out failure, resulting in higher dissipation of shear energy.

In addition, 38 mm polypropylene microfibers in this study recorded a higher fiber count in the given volume and improved the ability of the cementitious matrix in absorbing more shear energy, as compared to those of 54 mm. Nonetheless, the 19 mm nylon fibers were not able to impart higher shear strength than the polypropylene fibers, despite having higher tensile strength, due to the lower interfacial bonding between the fiber and cementitious matrix.

Furthermore, all HyFRC specimens in this study succeeded in improving the shear strength of conventional concretes with a change in the mode of failure from brittle to quasi-brittle. In addition, the developed HyFRC specimens, specifically the Ultra-Net (an increase of 32%), Super-Net (an increase of 24%), Econo-Net (an increase of 44%), and Nylo-Mono (an increase of 24%) hybrids, performed much better than the use of singular Ferro macro-sized fibers (FFCs) in concretes.

Last but not least, the Ultra-Net and Econo-Net HyFRC showed positive increases in their hybridization synergistic effects and enhanced performance in their shear energy absorption capabilities. However, the synergistic effects diminished with the widening of crack gaps due to the excessive dowel action imposed on the synthetic fibers, approaching the limitations of their ultimate tensile strength. On the other hand, Nylo-Mono hybrids produced negative synergistic energies at lower crack deflections due to the lower bonding properties of the fibers. Meanwhile, the Super-Net hybrids recorded negative deflection of 1 mm due to their fiber-bridging instability in the cementitious matrix. Nevertheless, positive synergistic effects were observed at larger deflections for the Ferro-Super and Ferro-Nylo hybrids, which demonstrated an increase in toughness with the widening of crack gaps.

## Figures and Tables

**Table 1:** Specifications of fibers as listed in the FORTA fibers technical data sheet. (UC: Ultra-Net; SC: Super-Net, EC: Econo-Net; NC: Nylo-Mono; FFC: Ferro).

Type	Length (mm)	Form	Bonding Power	Class	Material	Tensile Strength (MPa)
UC	54	Fibrillated Twisted Bundle	Extra heavy-duty	Micro	Polypropyleneand Additives	570–660
SC	38	Fibrillated	Heavy-duty			570–660
EC	38	Fibrillated	Medium-duty			570–660
NC	19	Monofilament	Light-duty		Virgin Nylon	966
FFC1	38	Fibrillated Twisted Bundle	Heavy-duty	Macro	Polyethylene, Polypropyleneand Additives	1100
FFC2	54		Heavy-duty			570–660

**Table 2:** HyFRC mix-design. (C: Control; U: Ultra-Net; S: Super-Net; E: Econo-Net; F: Ferro; Vol.: Volume).

Specimens	Designation	Type of Fibers (Vol. of Fraction, %)						Total Vol. Fraction, %
		Macrofibers		Microfibers				
		FF1	FF2	UN	SN	EN	NM	
Control	C (Plain)	-	-	-	-	-	-	-
Control	UC	-	-	0.30	-	-	-	0.30
Control	SC	-	-	-	0.30	-	-	0.30
Control	EC	-	-	-	-	0.30	-	0.30
Control	NC	-	-	-	-	-	0.30	0.30
Control	FFC	0.6	0.6	-	-	-	-	1.20
1	F4U2	0.4	0.4	0.20	-	-	-	1.00
2	F4U3	0.4	0.4	0.30	-	-	-	1.10
3	F6U2	0.6	0.6	0.20	-	-	-	1.40
4	F6U3	0.6	0.6	0.30	-	-	-	1.50
5	F4S2	0.4	0.4	-	0.20	-	-	1.00
6	F4S3	0.4	0.4	-	0.30	-	-	1.10
7	F6S2	0.6	0.6	-	0.20	-	-	1.40
8	F6S3	0.6	0.6	-	0.30	-	-	1.50
9	F4E2	0.4	0.4	-	-	0.20	-	1.00
10	F4E3	0.4	0.4	-	-	0.30	-	1.10
11	F6E2	0.6	0.6	-	-	0.20	-	1.40
12	F6E3	0.6	0.6	-	-	0.30	-	1.50
13	F4N2	0.4	0.4	-	-	-	0.20	1.00
14	F4N3	0.4	0.4	-	-	-	0.30	1.10
15	F6N2	0.6	0.6	-	-	-	0.20	1.40
16	F6N3	0.6	0.6	-	-	-	0.30	1.50
